# A Case of Erythræmia or Splenic Polycythæmia
1Reported at a meeting of the Medico-Chirurgical Society, April 14th, 1920.


**Published:** 1920-06

**Authors:** G. Parker

**Affiliations:** Physician to the Bristol General Hospital


### A CASE OF ERYTHRÆMIA OR SPLENIC POLYCYTHÆMIA.[1]

BY

G. Parker, M.D. Cantab.,

*Physician to the Bristol General Hospital.*

---

This is an uncommon disease first described by Vaquez of Paris in 1892, which has attracted a good deal of interest from the light it throws on the physiology of the circulation and upon the origin of the primary anæmias. Cases were described by Cabot, Osler, Saundby, Russell, Blumenthal, and others, so that L. E. J. Mackay was able to collect forty by 1907, and Parkes Weber wrote an admirable digest of the whole subject in 1910. It corresponds to what was vaguely described in the past as plethora, when, as the

[1] Reported at a meeting of the Medico-Chirurgical Society, April 14th, 1920.

popular phrase ran, all the patient's food turns to blood, the opposite, in fact, to anæmia.

Now polycythæmia exists when the number of red cells is above the normal average. It may be relative, as in cholera, where the water is drained off, or absolute when the erythrocytes and the whole mass of the blood is increased. Secondary forms of the latter occur both in men and animals at high altitudes, such as mountain plateaux, where an increase of erythrocytes of the mass of the blood are required to compensate for the deficiency of oxygen. They are found, too, in some cardiac and pulmonary diseases, notably in congenital heart cases, and in some forms of poisoning, *e.g.* phosphorus, CO., sulphonal, and acetanilide, where the oxygen capacity of the cells is low. With certain toxins which usually cause anæmia the reaction of the organism may cause polycythæmia, as occasionally in syphilis, malaria, tuberculosis, and paroxysmal hæmoglobinuria. Finally, it may occur in portal congestion, osteitis deformans, intermittent claudication, and some forms of gangrene.

The true primary polycythæmia or erythræmia has no known cause, resembling in this the primary anæmias, but in all the absolute polycythæmias there is an increased activity of the bone marrow, even nucleated, *i.e.* immature, erythrocytes may be found, and much of the marrow which is normally yellow will be red, showing the part it takes in cell formation. Thus erythræmia is marked by persistent absolute increase of the mass of the blood and erythrocytes, due to abnormal activity of the marrow. The erythrocytes may run up to even 11 or 13 millions, the hæmoglobin to 200, and a citrated tube of blood may show 9/10ths of its volume composed of red cells. The viscosity is raised and the mass of blood in the body may be two or three times the normal. There is usually cyanosis of the skin and fauces, and in a great proportion of the cases a hard, large spleen. The blood-pressure is often high, and urobilin and albumen may be present.

The details of my patient's condition are as follows :—

Mrs. S., aged 65, the mother of eleven children, was admitted to the Bristol General Hospital on February 13th, 1920, complaining of an abdominal swelling which caused some discomfort and breathlessness in going upstairs. It had been noticed for eighteen months. She has not been abroad, but underwent some mental trouble during the war. There had been no previous illness, except winter cough and bronchitis and some degree of constipation and flatulence. There had been no vomiting, hæmatemesis or other bleeding. The menopause occurred fifteen years ago, but for eighteen months attacks of flushing and giddiness were troublesome at times. She looked well and could not be called cyanotic, but had a fair amount of colour and the tongue was slightly dusky. The optic discs were normal. The urine once showed a trace of albumen, sp. gr. 1018, no sugar, a deposit of urates, no casts, no absorption bands of urobilin. There was nothing abnormal in the chest. The apex of the heart was in the 5th space, and internal to nipple-line, impulse not forcible, sounds and area of dulness normal. Blood-pressure=135, systolic. X-ray screen showed heart, aorta and lungs normal. The spleen was hard, movable, and enlarged, extending 2 inches below the umbilicus. The blood (March 29th, 1920) showed erythrocytes, 7 millions; hæmoglobin, 110; index, 0.89, red cells of good shape and staining reaction, one nucleated red cell seen; leucocytes, 4560 per c.m.; polymorphocytes, 68 per cent.; lymphocytes, 5.5 per cent.; large hyalines, 8 per cent.; transitional, 16.5 per cent.; eosinophiles, 1 per cent. I am not able to give the measure of the total volume of the blood or of its viscosity, but as I have said these are generally increased, and the work of the heart added to correspondingly.

Comparing her condition with that of other cases, while the condition is well marked, she is fortunate in not having severe symptoms at present. Thus there is little pigmentation of the face and hands. The strain on the heart from circulating a huge mass of blood with so many corpuscles is at present slight. She has a comparatively small increase of red cells compared with the 11 or 13 millions occasionally seen. She has no high blood-pressure or hemorrhages.

The prognosis is fair, and there may be good health for years, but later on ascites, cardiac failure and hemorrhages may be looked for.

Treatment by X-rays has been suggested, but few if any successes are reported. Saline purges are useful, and later on, when needed, repeated venesection and low diet will be indicated. Splenectomy appears to be harmful.

As to the causes of this condition, there is an excessive activity of the blood cell formation by the bone marrow, and we are reminded of the parallel case of the leucocythæmias, where there is an abnormal formation of white cells. Blumenthal, indeed, reported an instance where the two conditions were present, namely an excess of erythrocytes and of myelocytes. There is nothing suggesting such a state in my patient except the slight excess of large hyaline cells.

Various theories of the cause of the error of erythrocyte formation have been put forward, *e.g.* a reversion to the fœtal state or a neoplasm of the blood. Again, erythræmia has been ascribed to toxæmia, but no evidence for this has been forthcoming, or to the effects of obstructed circulation, though careful examinations have been made. The disease undoubtedly impedes the circulation, but is not caused by it as Lommel once thought. Nor is it due to an increased or prolonged life of the corpuscles so far as hæmolytic and other tests have shown. It is pretty clearly not due to diminished oxygen capacity of the blood such as occurs in CO. and other forms of poisoning. In short, we can only say that erythræmia is a disease of the bone marrow, but why this abnormal formation of red-blood cells takes place there is at present nothing whatever to show, though a similar process can be brought about in healthy persons by various poisons or by living at high altitudes.

